# Amino Acid-Based Hydrophobic Cryogels for Efficient Methylene Blue Removal: A Reusable and Eco-Friendly Approach to Dye-Contaminated Wastewater Treatment

**DOI:** 10.3390/gels11060411

**Published:** 2025-05-30

**Authors:** Merve Sofuoğlu, Ali Ender Kuyucu, Kadir Erol, Faruk Gökmeşe

**Affiliations:** 1 Department of Chemistry, Institute of Graduate Education, Hitit University, Corum 19030, Turkey; 2 Department of Chemistry, Institute of Science, Yüzüncü Yıl University, Van 65080, Turkey; 3 Department of Medical Services and Techniques, Health Services Vocational School, Hitit University, Corum 19030, Turkey; 4 Department of Chemistry, Faculty of Arts and Sciences, Hitit University, Corum 19040, Turkey

**Keywords:** adsorption, cryogel, dye, hydrophobic, methylene blue, phenylalanine

## Abstract

The release of synthetic dyes into the environment through industrial wastewater represents a significant environmental concern. In this study, a hydrophobic cryogel, Poly(2-hydroxyethyl methacrylate-N-methacryloyl-L-phenylalanine), was synthesized and employed for the efficient removal of methylene blue from aqueous solutions. The cryogel exhibited a surface area of 6.834 m^2^/g and a water retention capacity of 218.6%. Adsorption experiments conducted under various conditions revealed a high adsorption capacity of 1304.6 mg/g for MB. Thermodynamic analyses indicated that adsorption occurs spontaneously and follows a monolayer adsorption model. The adsorption capacity increased with temperature and ionic strength, confirming that hydrophobic forces predominantly drive the interaction. Reusability tests showed that the cryogel maintained its adsorption efficiency over five consecutive adsorption–desorption cycles, with a desorption efficiency of up to 98%. These findings demonstrate that Poly(HEMA-MAPA) cryogel is a practical, reusable, and eco-friendly adsorbent for removing methylene blue, a common textile dye pollutant, from water systems.

## 1. Introduction

Water pollution is a pressing global issue that affects ecosystems, public health, and access to clean water resources. Rapid industrialization, population growth, and climate change have exacerbated this problem, particularly in densely populated areas with high demand for clean water [1]. In response to these challenges, the reuse and recycling of wastewater have emerged as vital strategies for sustainable water management. However, even after conventional treatment, wastewater can still contain persistent organic pollutants that are difficult to remove completely [2].

Among these pollutants, synthetic dyes used extensively in various industries, especially textiles, pose a significant environmental and health risk due to their toxicity, resistance to degradation, and potential bioaccumulation [3]. Methylene blue (MB), a cationic heterocyclic aromatic dye with the molecular formula C_16_H_18_N_3_SCl, is one of the most commonly used dyes in the textile industry. In solid form, MB appears as an odorless dark green powder and dissolves in water to produce a characteristic blue solution [4,5]. It exhibits maximum absorbance in the visible spectrum at wavelengths between 660 and 670 nm [6].

Numerous physical, chemical, and biological methods have been developed to remove dyes from wastewater, including photodegradation, catalytic degradation, coagulation–flocculation, membrane separation, chemical oxidation, electro-flotation, solvent extraction, and reverse osmosis [7]. Despite their effectiveness, many of these techniques are either cost-prohibitive or generate secondary waste. In contrast, adsorption is widely recognized as one of the most efficient, economical, and straightforward methods for dye removal [8]. A broad range of adsorbent materials have been explored for this purpose, such as activated carbon [9], electrospun fibers [10], polymeric film [11], hydrogels [12], and, more recently, cryogels [13].

Hydrogels are three-dimensional, crosslinked hydrophilic polymer networks capable of absorbing and retaining large amounts of water due to their functional group (such as amide, carboxyl, or hydroxyl), which makes them attractive for wastewater treatment applications [14,15]. Cryogels, a special class of hydrogels, are synthesized through cryo-polymerization at sub-zero temperatures. Upon thawing, they form interconnected macroporous structures that provide high permeability and rapid mass transfer, making them particularly suitable for applications in drug delivery [16], tissue engineering [17], bone regeneration [18], and cell preservation [19]. In recent years, cryogels have gained attention in the field of environmental remediation, especially for water purification [20].

Despite extensive efforts to develop advanced adsorbents for dye removal, most current materials lack sufficient selectivity, rapid uptake, and reusability, particularly under challenging aqueous conditions. This study introduces a novel cryogel system based on N-methacryloyl-L-phenylalanine (MAPA). This hydrophobic and aromatic amino acid-derived monomer [21,22] has not previously been applied for dye remediation. Incorporating MAPA into the 2-hydroxyethyl methacrylate (HEMA) matrix enables enhanced π–π interactions and hydrophobic binding with MB, a commonly used aromatic dye [23]. Various cryogel-based systems have recently been explored to remove MB from aqueous media. For instance, Yin et al. developed alginate/poly(styrenesulfonate) cryogels with a maximum MB adsorption capacity of 2300 mg/g [7], while Meneses et al. utilized CTAB-modified cryogels for simultaneous MB and heavy metal removal [8]. Additionally, chitosan-based cryogels functionalized with glycidyl methacrylate demonstrated effective MB binding under mild conditions [13]. These examples underscore the growing interest in cryogels for dye remediation and highlight the importance of material-specific functionalization strategies, such as our MAPA-based approach. While conventional cryogels often depend on electrostatic interactions for dye removal, such mechanisms can be sensitive to pH and ionic strength. In contrast, hydrophobic interactions offer a more robust and selective binding strategy, particularly for dyes like MB that contain planar aromatic structures. This hydrophobic functionalization strategy remains underexplored in the literature, thus providing a novel perspective for developing high-performance cryogel-based adsorbents.

## 2. Results and Discussion

### 2.1. Characterization

**Figure 1:**
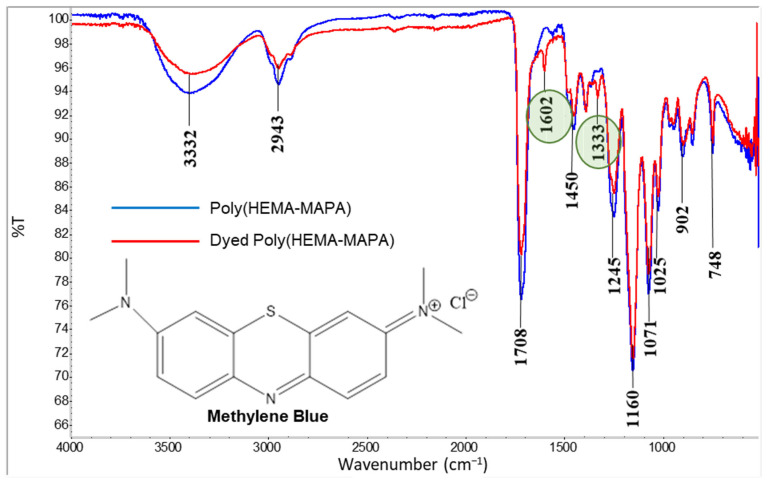
FT-IR spectra of bare and dye-adsorbed Poly(HEMA-MAPA) cryogels.

FTIR spectroscopy was employed to express the chemical structure of the Poly(HEMA-MAPA) cryogel (Figure 1). A broad and intense peak observed at 3332 cm^−1^ corresponds to the O–H stretching vibrations, indicating the presence of hydroxyl groups. The peak at 2943 cm^−1^ is attributed to asymmetric C–H stretching vibrations. A distinct absorption band appearing at approximately 1708 cm^−1^ is assigned to the C=O stretching vibrations characteristic of carbonyl functionalities. Moreover, the disappearance of the characteristic C=C stretching vibration (typically observed around 1635–1640 cm^−1^ in monomers) confirms the successful polymerization of the methacrylate double bonds in HEMA and MAPA. Around 1450 cm^−1^, the band is linked to N–H bending vibrations (amide group). Characteristic absorption bands were observed at 1245 cm^−1^ (C–O stretching of ester groups), 1160 cm^−1^ (C–O–C asymmetric stretching), 1025 cm^−1^ (overlapping C–N and C–O stretching), and 902 cm^−1^ (C–H out-of-plane bending of aromatic rings), confirming the presence of ester, ether, amide, and aromatic functionalities in the cryogel structure.

Additionally, aromatic ring vibrations originating from the MAPA monomer are evidenced by the bands at 1071 and 748 cm^−1^ [24] (Figure S1). In the FTIR spectrum of the dyed cryogel, a prominent band at 1602 cm^−1^, absent in the undyed sample, corresponds to the C=N stretching vibrations in the central ring. Furthermore, the band at 1333 cm^−1^ is ascribed to the C=S stretching vibration in the heterocyclic ring, confirming the interaction of the dye with the cryogel matrix [25] (Figure 1).

**Figure 2:**
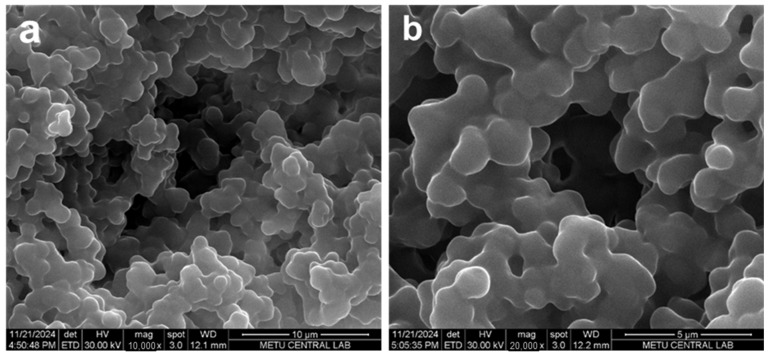
SEM images of Poly(HEMA-MAPA) cryogel. (**a**) Low-magnification image (10,000×) showing the highly porous and interconnected 3D network structure typical of cryogel matrices. (**b**) Higher magnification image (20,000×) revealing the wrinkled and sponge-like surface morphology with open pores, facilitating enhanced diffusion and surface accessibility.

SEM images (Figure 2a,b) clearly reveal that the synthesized Poly(HEMA-MAPA) cryogel exhibits a highly macroporous structure with an interconnected porous network. The slight agglomeration observed in the SEM images may result from freeze-drying or uneven polymerization, which could be minimized by optimizing surfactant use or applying ultrasonic dispersion. The cryogel matrix comprises irregularly shaped and densely packed polymeric granules forming numerous flow channels and void spaces, facilitating the rapid diffusion of dye molecules during adsorption. These morphological characteristics are critical for applications requiring high adsorption performance.

In particular, the structure’s rough and uneven surface topography significantly enhances the specific surface area available for interaction with target molecules. This roughness plays a vital role in adsorption processes by providing more active binding sites and by increasing the contact time between adsorbate and adsorbent, which aligns with the principles of collision theory. According to this theory, a sufficient surface roughness increases the likelihood of effective collisions and interactions between dye molecules and the adsorbent surface, thereby improving overall adsorption efficiency [26]. Moreover, at higher magnifications (Figure 2b), the interconnected pores ranging from several microns to sub-micron sizes become more apparent, confirming the open-channel architecture that supports convective mass transport and reduces diffusion limitations. Such a structure improves kinetic performance and allows for multiple adsorption–desorption cycles, which is beneficial for material reusability in environmental remediation applications [27].

Elemental analysis revealed that the nitrogen content of the Poly(HEMA-MAPA) cryogel was approximately 0.47% by weight. This nitrogen originates from the amide group of the MAPA monomer which contains a single nitrogen atom per molecule. Considering the molecular weight of MAPA (~233.11 g/mol) and its nitrogen content (6.01%), a stoichiometric calculation was performed to determine the extent of MAPA incorporation into the polymer. Based on this approach, the amount of MAPA integrated into the polymer structure was calculated to be 335.5 µmol per gram of polymer. Given the initial feed ratio of 22 mmol HEMA and 0.214 mmol MAPA, the resulting copolymer composition corresponds to an approximate HEMA:MAPA molar ratio of 66:1, suggesting partial MAPA incorporation during polymerization. This result confirms the successful functionalization of the cryogel with MAPA units, enhancing its potential for selective adsorption through amide and aromatic interactions.

The swelling capacity of the Poly(HEMA-MAPA) cryogel was assessed to evaluate its water absorption capability, which is a critical parameter for adsorption-based applications. As a result of the swelling test, the cryogel demonstrated an average water retention rate of 218.6% (n = 3). This finding indicates that 1 g of dry cryogel can absorb and retain approximately 2.19 g of water within its porous matrix. Such a high swelling ratio reflects the presence of a highly porous, hydrophilic network that facilitates efficient water uptake and the diffusion of target molecules throughout the cryogel structure. Incorporating MAPA significantly enhanced the hydrophobicity and functional group diversity of the cryogel matrix. The observed water retention of 218.6% is within the typical range for cryogels designed for aqueous-phase applications (100–300%) [28].

In addition to its swelling performance, the specific surface area of the cryogel was determined to be 6.834 m^2^/g, as analyzed by BET surface area analysis (Figure S2). The surface area of macroporous cryogels typically ranges between 1 and 10 m^2^/g, which is considered relatively high for such materials. Therefore, this surface area is sufficient to provide accessible active sites for interactions with adsorbates, despite being lower than that of activated carbon-based adsorbents [29]. The combination of high water retention and notable surface area suggests that Poly(HEMA-MAPA) cryogel provides an effective platform for adsorption studies, particularly in aqueous media, where rapid mass transfer and structural stability are essential [30].

### 2.2. Adsorption Studies

**Figure 3:**
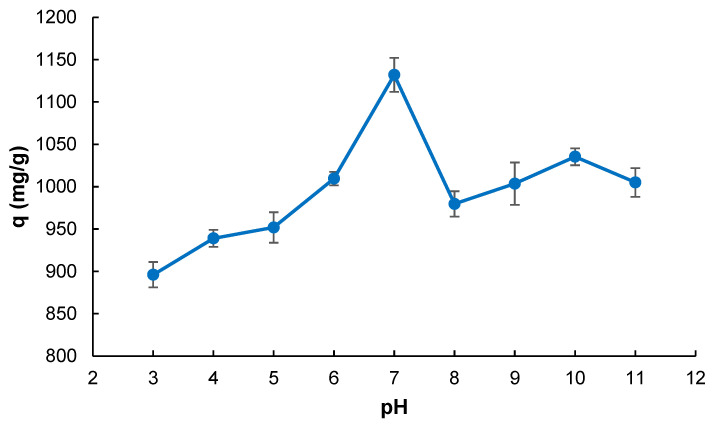
pH effect on MB adsorption (*V*: 4 mL; *C*_0_: 1000 mg L^−1^; *T*: 298 K; *t*: 60 min).

Adsorption studies were conducted across a broad pH range (3.0–11.0) to evaluate the impact of solution pH on the adsorption capacity of the cryogel towards methylene blue (MB). As depicted in Figure 3, the adsorption capacity exhibited a clear dependence on pH, reaching its maximum at pH 7.0. The sharp increase in adsorption at pH 7.0 is attributed to the optimal balance between the surface charge neutrality of the cryogel and dye ionization, which facilitates enhanced hydrophobic and π–π stacking interactions while minimizing electrostatic repulsion [31]. Moreover, zeta potential analysis (Figure S3) revealed that the isoelectric point of the cryogel is approximately pH 6.95. Below this pH, the cryogel surface is positively charged, leading to potential electrostatic repulsion of the cationic MB dye (pKb ≈ 5.2). Above the IEP, the cryogel surface becomes negatively charged, favoring electrostatic attraction. This transition explains the observed enhancement in adsorption capacity near pH 7.0, where electrostatic repulsion is minimized, and hydrophobic and π–π interactions are maximized. Phenylalanine residues incorporated into the polymer network possess hydrophobic aromatic side chains that can interact with the aromatic rings of MB via π–π stacking and hydrophobic interactions. At highly acidic or basic pH levels, the ionization of both the MB molecules and the functional groups on the cryogel surface may promote electrostatic repulsion or competition, thereby reducing the efficiency of hydrophobic interactions. Thus, the optimal adsorption observed at pH 7.0 suggests a favorable balance of interaction forces, supporting the proposed mechanism based predominantly on hydrophobic effects [32,33].

**Figure 4:**
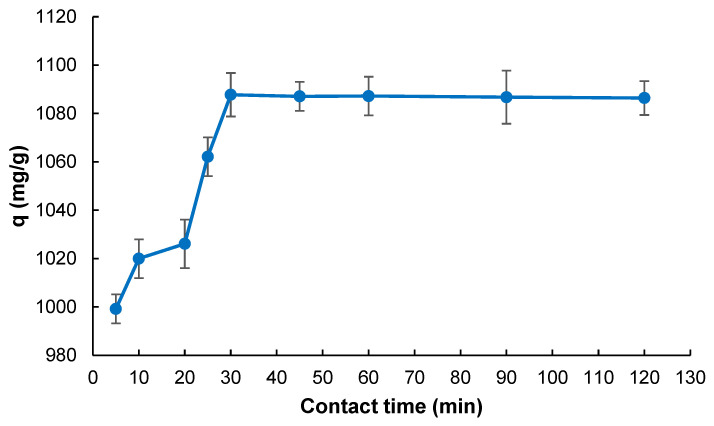
Effect of contact time on MB adsorption (*V*: 4 mL; *C*_0_: 1000 mg L^−1^; *T*: 298 K; *pH*: 7.0).

Figure 4 illustrates the effect of contact time on the adsorption capacity of the cryogel for MB. As can be seen, the adsorption capacity increases significantly during the initial phase of the process, particularly within the first 30 min. This sharp increase can be attributed to the abundant availability of active adsorption sites on the cryogel surface, which enables the rapid uptake of dye molecules via mechanisms such as electrostatic attraction and hydrophobic interactions.

Beyond 30 min, the adsorption curve reaches a plateau, indicating that the adsorption process approaches equilibrium. This stagnation suggests that most of the available adsorption sites have been occupied, and the cryogel matrix has reached a saturation point with MB molecules. Therefore, no substantial increase in adsorption capacity is observed with further prolongation of contact time up to 120 min. The slight fluctuations in *q* values after equilibrium may be associated with minor desorption or surface rearrangement events. Still, these are within the range of experimental error, as indicated by the error bars.

Thus, the optimal contact time for efficient adsorption was determined to be 30 min, beyond which extending the contact time does not significantly enhance adsorption efficiency. This result aligns with previous findings reported in the literature, confirming that most dye uptake occurs rapidly in the early stages of contact due to high concentration gradients and unsaturated active sites [34]. Such kinetics are favorable in practical applications where the rapid removal of contaminants is desired, offering time-efficient treatment performance.

**Figure 5:**
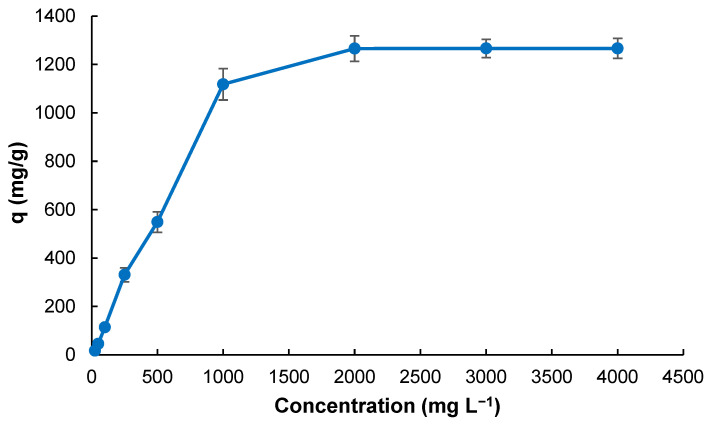
Effect of dye concentration on MB adsorption (*V*: 4 mL; *T*: 298 K; *t*: 30 min; *pH*: 7.0).

Figure 5 presents the effect of the initial MB concentration on the adsorption capacity of the Poly(HEMA-MAPA) cryogel. As illustrated, the adsorption capacity increases markedly with increasing dye concentration in the range of 25–2000 mg L^−1^. The greater driving force for mass transfer between the bulk solution and the cryogel surface at higher solute concentrations can explain this initial rise. The available MB molecules are limited at low concentrations, and the adsorption sites are in excess; hence, the driving force for adsorption is limited due to the low concentration of dye molecules. As the concentration increases, more MB molecules interact with the active sites on the cryogel, leading to a significant increase in *q*.

Beyond the concentration of approximately 2000 mg L^−1^, a plateau is observed in the adsorption capacity, suggesting that the cryogel has reached its saturation point. This implies that the available interaction zones, including hydrophilic, hydrophobic, and electrostatic binding domains provided by the functional MAPA groups, are fully occupied by MB molecules. The lack of further increase in *q* with rising MB concentrations indicates that the adsorption sites have become limited, and no additional dye molecules can be effectively retained.

This saturation behavior is consistent with the monolayer adsorption theory and supports the hypothesis that the binding sites on the Poly(HEMA-MAPA) cryogel are finite and specific. The maximum adsorption capacity was observed at 2000 mg L^−1^, indicating the saturation limit of the cryogel under the tested conditions. However, the material also demonstrated high adsorption efficiency at lower dye concentrations. The stability of *q* values beyond this point also demonstrates the robustness of the cryogel structure under high MB loading conditions, which is desirable for potential applications in high-strength industrial wastewater treatment.

These findings corroborate prior studies in which the increase in adsorbate concentration enhanced adsorption until the adsorbent reached equilibrium saturation [35].

**Figure 6:**
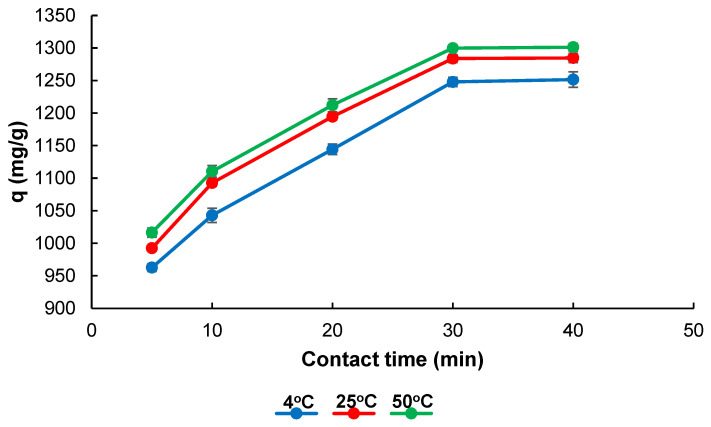
Effect of temperature on adsorption capacity at different contact times (*V*: 4 mL; *C*_0_: 2000 mg L^−1^; *pH*: 7.0).

Figure 6 illustrates the contact time and temperature effect on the MB adsorption capacity onto the Poly(HEMA-MAPA) cryogel. As observed, the adsorption capacity increased significantly within the first 30 min across all temperatures studied (4 °C, 25 °C, and 50 °C), indicating rapid initial uptake due to the abundance of accessible active sites on the cryogel surface. Beyond 30 min, the adsorption rate plateaus, suggesting that equilibrium has been reached and the available binding sites are saturated with MB molecules.

A closer data comparison reveals a distinct temperature-dependent trend: higher temperatures correspond to higher adsorption capacities at each time point. For example, at 30 min, the adsorption capacity reaches approximately 1320 mg/g at 50 °C, compared to about 1300 mg/g at 25 °C and 1250 mg/g at 4 °C. This trend strongly implies that the adsorption mechanism is endothermic in nature and facilitated by increasing thermal energy [36].

The enhanced adsorption at elevated temperatures can be attributed to the entropy-driven nature of hydrophobic interactions between the MB molecules and the hydrophobic MAPA moieties incorporated in the cryogel structure. As temperature rises, the system’s entropy increases, amplifying the strength of the hydrophobic interactions [37]. Such behavior is characteristic of systems in which non-polar interactions play a dominant role in binding processes. Therefore, the data support the hypothesis that hydrophobic forces significantly contribute to the MB adsorption mechanism onto Poly(HEMA-MAPA) cryogels [38]. These findings not only validate the thermodynamic favorability of the process, but also reinforce the potential applicability of the cryogel material in dye removal under a range of environmental temperatures.

**Figure 7:**
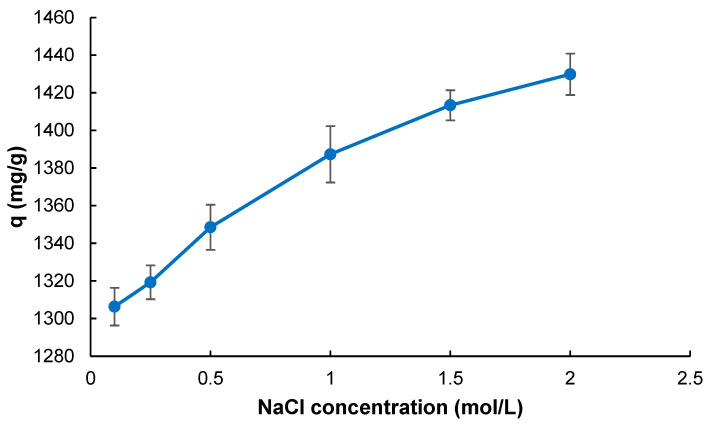
The effect of ionic strength on adsorption capacity (*V*: 4 mL; *C*_0_: 2000 mg L^−1^; *T*: 298 K; *t*: 30 min; *pH*: 7.0).

Figure 7 demonstrates the influence of ionic strength on MB adsorption capacity onto Poly(HEMA-MAPA) cryogels. NaCl was used to vary the ionic strength from 0.1 to 2.0 M systematically. As the concentration of NaCl increases, the adsorption capacity also shows a gradual and significant enhancement, reaching approximately 1440 mg/g at 2.0 M NaCl compared to about 1300 mg/g in the absence of salt.

The fundamental principles of hydrophobic interaction enhancement in high-ionic-strength environments can explain this trend. In aqueous solutions, hydrophobic interactions are often masked by structured water molecules forming hydration shells around non-polar regions. However, adding neutral salts like NaCl disrupts these structured water networks. The salt ions compete for water molecules, effectively reducing the solvation of hydrophobic domains and leading to the so-called “salting-out” effect [39].

As a result, the hydrophobic groups in the MB molecules and on the MAPA-functionalized cryogel surfaces are brought closer due to decreased hydration, thereby increasing the likelihood of hydrophobic interactions. This behavior is well documented in hydrophobic interaction chromatography (HIC), where salt-enhanced binding is commonly utilized to promote ligand–biomolecule interactions [40]. In this context, the increasing adsorption capacity with rising NaCl concentration suggests that hydrophobic interactions play a dominant role in the MB adsorption mechanism. It also confirms that the ionic strength of the medium is a key tunable parameter for optimizing the performance of cryogel-based adsorbents in dye removal applications.

**Figure 8:**
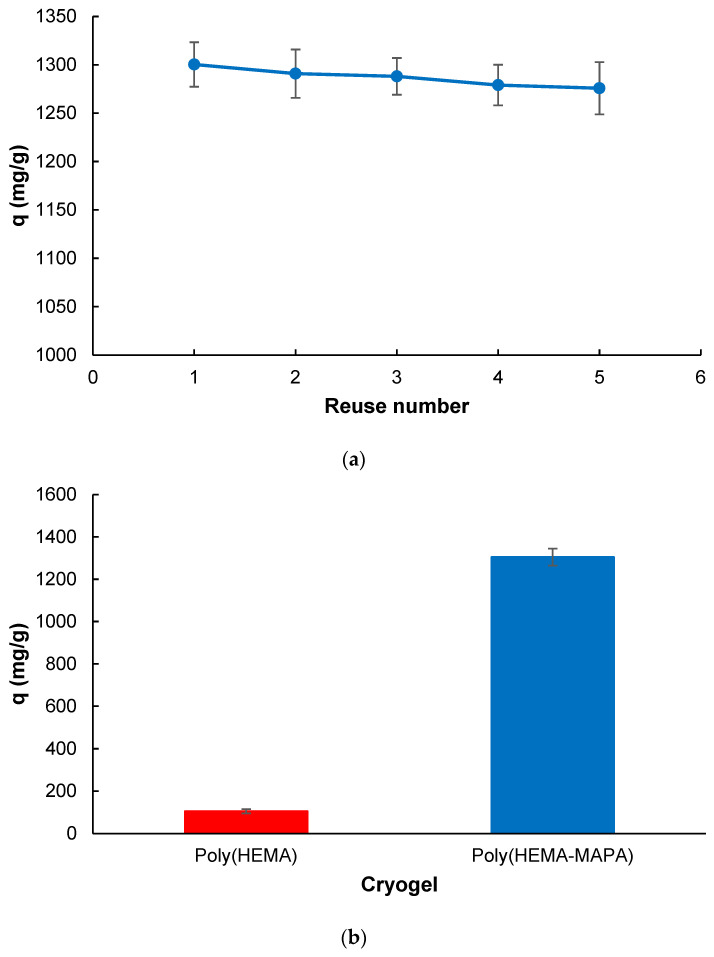
(**a**) Reusability of poly(HEMA-MAPA) cryogel. (**b**) Comparison of MB adsorption performances of Poly(HEMA) and Poly(HEMA-MAPA) cryogels (*V*: 4 mL; *C*_0_: 2000 mg L^−1^; *T*: 298 K; *t*: 30 min; *pH*: 7.0).

Figure 8a illustrates the change in adsorption capacity of the cryogels over five consecutive adsorption–desorption cycles. The results demonstrate that the cryogels maintained their performance with only a negligible decline in adsorption capacity, from approximately 1320 mg/g in the first cycle to around 1280 mg/g in the fifth. The cryogels exhibited excellent structural and operational stability, maintaining over 98% of their initial adsorption capacity after five consecutive adsorption–desorption cycles. No visible deformation, mass loss, or surface collapse was observed throughout these cycles, indicating strong mechanical integrity and reusability. This stable performance confirms the robustness of the Poly(HEMA-MAPA) matrix under repetitive use conditions and supports its potential for practical wastewater treatment applications. The cryogels represent a promising, reusable, and environmentally friendly adsorbent for dye removal processes [41].

Figure 8b compares the adsorption capacities of Poly(HEMA) and Poly(HEMA-MAPA) cryogels for MB under identical experimental conditions to assess the influence of MAPA functionalization. The data clearly show a dramatic enhancement in adsorption performance upon MAPA incorporation. Poly (HEMA-MAPA) achieved an adsorption capacity exceeding 1300 mg/g, while Poly(HEMA) exhibited only around 8.1% of this value. The minimal adsorption observed for Poly(HEMA) is likely attributed to weak interactions between the hydroxyl (-OH) groups of HEMA and the MB molecules. In contrast, the significant increase in adsorption for Poly(HEMA-MAPA) highlights the pivotal role of MAPA in introducing specific functional groups, such as amine or hydrophobic moieties, that facilitate stronger interactions with the MB dye through electrostatic and hydrophobic interactions. These findings demonstrate the effectiveness of MAPA modification in enhancing dye-binding capacity, underscoring its critical contribution to developing high-performance adsorbent systems. Considering that many conventional cryogels exhibit capacities below 500 mg/g [42], the performance of Poly(HEMA-MAPA) indicates a strong potential for practical dye removal applications.

### 2.3. Isotherm Studies

To gain a deeper insight into the nature and mechanism of MB adsorption onto Poly(HEMA-MAPA) cryogels, three adsorption isotherm models—Langmuir, Freundlich, and Flory–Huggins—were applied to the experimental data. Among these models, the Langmuir isotherm exhibited the best fit with the experimental data, with a high correlation coefficient (R^2^ = 0.9706) (Table 1). The theoretical maximum adsorption capacity (*Q_max_* = 1250 mg/g) obtained from this model closely matched the experimental value (1304.6 mg/g), indicating that the adsorption process occurs via a monolayer mechanism on a homogeneous cryogel surface with energetically uniform active sites.

This finding suggests that each adsorption site interacts with a single MB molecule, without lateral interactions between the adsorbed molecules. The small value of the Langmuir constant *b* (0.000093 L mg^−1^) further indicates moderate affinity, characteristic of physisorption processes accompanied by non-covalent forces, such as hydrophobic and electrostatic interactions. The low values observed in the Freundlich model (R^2^ = 0.6877) suggest a limited degree of surface heterogeneity or site diversity, reinforcing the dominance of monolayer adsorption [35].

The Flory–Huggins model was also employed to assess the feasibility and spontaneity of the adsorption process. The negative Gibbs free energy changes (Table 2) derived from the Flory–Huggins equilibrium constant (*K_FH_* = 0.00015) confirm the thermodynamically favorable and spontaneous nature of MB adsorption onto the cryogel surface [43].

Thermodynamic parameters were calculated using Van’t Hoff plots to complement the isotherm analysis. A linear trend was obtained by plotting *lnK* versus 1/*T* using equilibrium data collected at different temperatures (277 K, 298 K, and 323 K). The slope and intercept of the plot yielded an enthalpy change (Δ*H*°) of +36.57 kJ mol^−1^ and an entropy change (Δ*S*°) of +196.6 J mol^−1^ K^−1^, respectively (Table 2).

The positive Δ*H*° value indicates that the adsorption is endothermic, which favors the process at elevated temperatures. This agrees with the experimental observations showing enhanced MB uptake at 50 °C. The positive Δ*S*° value suggests increased randomness at the solid–liquid interface during the adsorption process. This entropy gain may be attributed to the desolvation of MB molecules and rearrangement of structured water molecules on the cryogel surface during dye adsorption, a common characteristic of hydrophobic interactions [44]. These results confirm that MB adsorption onto Poly(HEMA-MAPA) cryogels is spontaneous, thermodynamically favorable, and predominantly driven by hydrophobic interactions under monolayer conditions.

### 2.4. Kinetic Modeling

Kinetic modeling was performed to better understand the adsorption mechanism and rate-limiting steps involved in the MB removal process using Poly(HEMA-MAPA) cryogels. Experimental data were evaluated using two widely adopted models: pseudo-first-order and pseudo-second-order kinetic models (Table 3).

The pseudo-first-order model assumes that the rate of occupation of adsorption sites is proportional to the number of unoccupied sites. However, the model yielded a relatively low correlation coefficient (R^2^ = 0.8546) and a calculated equilibrium adsorption capacity (*q_e_* = 1066.1 mg/g) that significantly deviated from the experimental value (1304.6 mg/g). This mismatch suggests that the pseudo-first-order model inadequately describes the adsorption process.

In contrast, the pseudo-second-order kinetic model provided a much better fit to the experimental data, with an excellent correlation coefficient (R^2^ = 0.9985) and a calculated *q_e_* value (1250.0 mg/g) very close to the experimental value. This implies that chemisorption is the dominant rate-controlling mechanism, involving valence forces through sharing or exchanging electrons between MB molecules and functional groups on the cryogel surface [45].

Furthermore, the pseudo-second-order kinetics also support the hypothesis that the macroporous structure of the cryogel enables the rapid diffusion and efficient interaction of MB molecules with the functional MAPA groups. These groups provide both hydrophobic phenyl rings and amide functionalities, which enhance dye affinity via π–π stacking and electrostatic interactions [46]. Furthermore, the applicability of this model indicates that the adsorption process is not limited by film diffusion or intraparticle diffusion, owing to the high porosity and interconnected channels of the cryogel matrix. Thus, the cryogel ensures efficient accessibility to binding sites, enabling fast kinetics and high adsorption capacity [29].

In summary, the kinetic results reveal that the adsorption of MB onto Poly(HEMA-MAPA) cryogels is governed by a chemically controlled process that follows pseudo-second-order kinetics. This process offers rapid uptake and high efficiency, ideal for practical water treatment applications [38].

### 2.5. Comparison with the Literature

MB adsorption has garnered significant attention in recent years due to its widespread use in textile industries and its potential environmental hazards. Numerous studies have investigated various cryogel-based adsorbents for removing MB from aqueous solutions. A comparative overview of these studies is summarized in Table 4, highlighting the adsorption method, isotherm model, maximum adsorption capacity, and equilibrium time for different cryogel systems.

Among the listed materials, Poly(HEMA-MAPA) cryogel demonstrated an impressive adsorption capacity of 1304.6 mg/g, with a notably short equilibrium time of just 30 min. When compared to other cryogels, such as AlgMA/PNaSS (2300 mg/g, 480 min) and Poly(ionic liquids) (1228.8 mg/g, 10 min), Poly(HEMA-MAPA) offers a competitive balance between high adsorption capacity and rapid kinetics. While AlgMA/PNaSS exhibits the highest capacity overall, it requires a significantly longer time to reach equilibrium, which may limit its practical applicability in high-throughput systems. On the other hand, although Poly(ionic liquid) reaches equilibrium quickly, its slightly lower adsorption capacity than Poly(HEMA-MAPA) suggests that it may not be as efficient in systems requiring higher uptake.

In comparison to traditional cryogels such as Poly(itaconic acid) and SA/clay quasi, which exhibit capacities of 172.4 and 181.8 mg/g, respectively, Poly(HEMA-MAPA) represents a substantial improvement in both adsorption performance and time efficiency. Furthermore, the adsorption behavior of Poly(HEMA-MAPA) fitting in the Langmuir isotherm model suggests a monolayer adsorption mechanism on a homogeneous surface, indicating the presence of uniform binding sites contributed by MAPA functional groups.

These findings collectively underscore the potential of Poly(HEMA-MAPA) cryogels as effective adsorbent materials for MB removal. The combination of high adsorption capacity, fast equilibrium, and straightforward batch application makes this material particularly attractive for practical wastewater treatment applications.

### 2.6. MB Adsorption from Real Wastewater Sample

**Figure 9:**
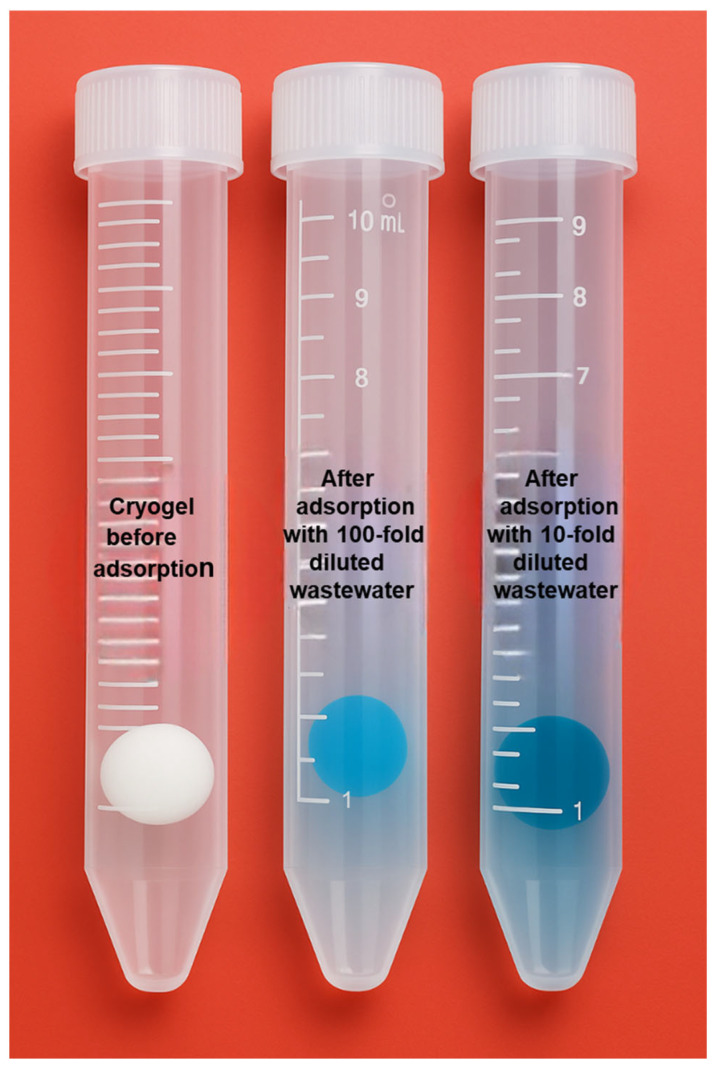
Images of cryogel before and after adsorption studies with real wastewater samples.

In this experimental study, 400 µL of real wastewater sourced from a textile manufacturing facility was diluted at a ratio of 1:10 and 1:100 with ultrapure water to obtain a final volume of 4 mL (Figure 9). An adsorption experiment was then conducted under optimized conditions for 30 min using the Poly(HEMA-MAPA) cryogel. As a result, the adsorption capacity was determined to be 813.8 mg/g.

Although this value is relatively high, it is notably lower than the maximum adsorption capacity observed with the synthetic methylene blue (MB) solution prepared under controlled laboratory conditions. This discrepancy is primarily attributed to the complex chemical matrix of real wastewater, which contains not only MB, but also various co-existing dye molecules, organic compounds, metal ions, and other interfering species. These contaminants can compete with MB molecules for active adsorption sites on the cryogel surface or may block access to these sites, thereby reducing the overall adsorption efficiency for MB.

Such interference effects are common in real-world applications and highlight the importance of evaluating adsorbents in ideal conditions and under practical scenarios. Despite the presence of interfering substances, the Poly(HEMA-MAPA) cryogel still demonstrated a strong adsorption performance, reinforcing its potential for efficient dye removal from complex industrial effluents.

## 3. Conclusions

This study demonstrated the successful synthesis and application of a novel hydrophobic cryogel, Poly(HEMA-MAPA), to efficiently remove methylene blue (MB) from aqueous solutions. The cryogel exhibited a high surface area and swelling capacity, facilitating rapid and high-capacity adsorption, with a maximum MB uptake of 1304.6 mg/g achieved within 30 min. Adsorption behavior was best described by the Langmuir isotherm and pseudo-second-order kinetic models, indicating monolayer coverage and chemisorption mechanisms. Thermodynamic evaluations confirmed that adsorption is spontaneous and endothermic, primarily driven by hydrophobic interactions between MAPA-functionalized cryogel surfaces and MB molecules. In agreement with the thermodynamic results, increased adsorption at higher temperatures and salt concentrations further supported the dominance of entropy-favored hydrophobic binding. Additionally, the cryogel maintained over 98% of its adsorption efficiency after five consecutive reuse cycles, highlighting its structural stability and practical reusability. Notably, the cryogel also showed promising adsorption performance in real textile wastewater samples, despite competitive interference from coexisting species. Compared with other cryogel-based adsorbents, Poly(HEMA-MAPA) displayed a favorable balance of high adsorption capacity and rapid kinetics, reinforcing its potential for real-world dye remediation. Overall, this work introduces a robust and reusable cryogel system that integrates functional hydrophobic monomers for enhanced dye binding, offering a sustainable and efficient approach to industrial wastewater treatment.

## 4. Materials and Methods

### 4.1. Materials

Sigma (St. Louis, MO, USA) provided the following: methylene blue (MB), 2-hydroxyethyl methacrylate (HEMA), ethylene glycol dimethacrylate (EGDMA), ammonium persulfate (APS), sodium lauryl sulphate (SLS), N,N,N′,N′-tetramethylethylene diamine (TEMED), and L-phenylalanine. No further purification or treatment was applied to the chemicals; they were used exactly as supplied. N-methacryloyl-l-phenylalanine (MAPA) compound was synthesized in a laboratory environment.

### 4.2. Methods

#### 4.2.1. Synthesis of MAPA

**Figure 10:**
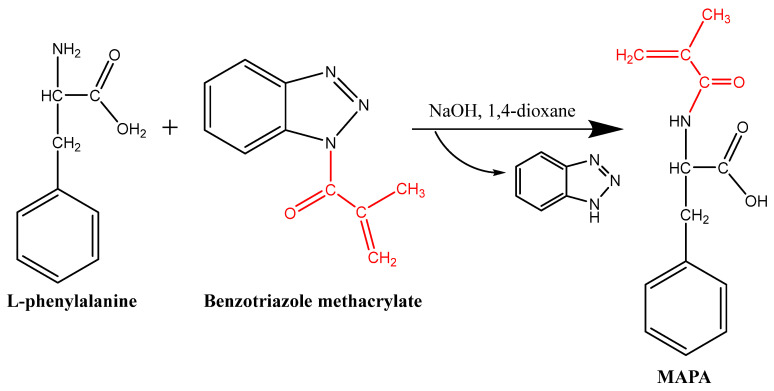
Synthesis of the MAPA monomer.

MAPA was synthesized following a previously reported method with slight modifications [51]. Initially, 1.0 g of L-phenylalanine was dissolved in 25 mL of 1 M aqueous sodium hydroxide solution. In a separate flask, 1.033 g of benzotriazole methacrylate was dissolved in 25 mL of 1,4-dioxane. The resulting solution was added dropwise to the phenylalanine solution under continuous stirring at room temperature. The reaction mixture was stirred for an additional 20 min to ensure complete reaction. Following the reaction, 1,4-dioxane was removed under reduced pressure using a rotary evaporator. The resulting precipitate was washed thoroughly with distilled water to eliminate any unreacted benzotriazole. The aqueous phase was subsequently extracted three times with 50 mL portions of ethyl acetate. After phase separation, the aqueous layer was neutralized to pH 6.0–7.0 using a 10% hydrochloric acid solution. Finally, evaporation was used to remove the water, yielding the MAPA compound as the final product (Figure 10).

#### 4.2.2. Synthesis of Poly(HEMA-MAPA) Cryogel

**Figure 11:**
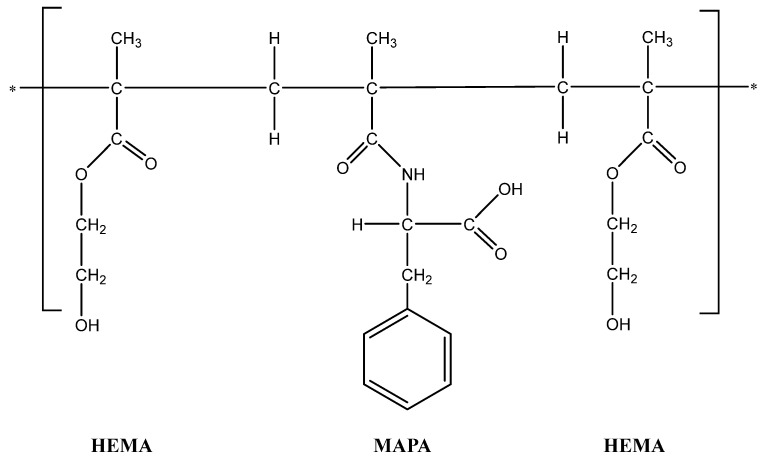
Structure of poly(HEMA-MAPA) cryogel. The asterisk (*) denote the continuation of the polymer chain, indicating the repeating nature of the copolymer composed of HEMA and MAPA units.

To synthesize the Poly(HEMA-MAPA) cryogel, 50 mg of MAPA, 2.5 mL of HEMA, and 2.5 mL of distilled water were initially mixed. Subsequently, 0.5 g of sodium lauryl sulfate (SLS) was added as a surfactant, followed by 0.6 mL of ethylene glycol dimethacrylate (EGDMA) as a crosslinking agent. The final volume of the mixture was adjusted to 15 mL with distilled water. The solution was stirred using a magnetic stirrer until a homogeneous mixture was obtained, then placed in an ice bath for 15–20 min to stabilize the system. Polymerization was initiated by adding 10 mg of ammonium persulfate (APS) and 50 μL of N,N,N′,N′-tetramethylethylenediamine (TEMED). The resulting mixture was poured between glass plates and allowed to polymerize at −12 °C for 24 h (Figure 11). Upon completion, the formed cryogels were cut into membrane (disc) shapes and washed with distilled water under agitation until foaming ceased and the wash water became clear, ensuring the removal of unreacted components and surfactant residues [52].

#### 4.2.3. Synthesis of Poly(HEMA) Cryogel

For comparison purposes, a Poly(HEMA) cryogel without the MAPA monomer was also synthesized. The procedure was identical to the Poly(HEMA-MAPA) cryogel synthesis (Section 4.2.2), except that no MAPA was added. Briefly, 2.5 mL of HEMA and 0.5 g of SLS were mixed with 2.5 mL of distilled water and 0.6 mL of EGDMA as a crosslinker. After thorough mixing and cooling in an ice bath, polymerization was initiated by adding 10 mg of APS and 50 μL of TEMED. The mixture was allowed to polymerize at −12 °C for 24 h. The resulting Poly(HEMA) cryogels were washed and dried using the previously described protocol.

### 4.3. Characterization of Poly(HEMA-MAPA) Cryogel

#### 4.3.1. Swelling Test

The water retention capacity of the Poly(HEMA-MAPA) cryogels was evaluated using a swelling test in distilled water. Initially, the samples were dried using lyophilization to preserve their macroporous structure. Freeze-drying minimizes capillary stress, maintaining the cryogel’s internal architecture and ensuring consistent sorption and swelling behavior. Then, a dry cryogel membrane was carefully weighed (*W*_0_) and immersed in distilled water maintained at 25 °C in a constant-temperature water bath. After 30 min of swelling, the membrane was gently removed, blotted with filter paper to eliminate surface water, and reweighed (*W_s_*). The water retention capacity (%) was calculated using the following equation:

(1) $$\mathrm{Water}\;\mathrm{retention}\;\mathrm{capacity}\;\%=[(W_{s}-W_{0})/\;W_{0}]\times 100$$

Here, *W*_0_ is the initial weight of the dry cryogel and *W_s_* is the weight of the swollen cryogel after immersion. This test provides insight into the cryogel’s ability to absorb and retain water, which is critical for evaluating its suitability in aqueous adsorption applications.

#### 4.3.2. Surface Morphology Analysis

The surface morphology of the synthesized cryogels was examined using scanning electron microscopy (SEM). Before imaging, the cryogel samples were lyophilized to remove moisture and preserve structural integrity. A sufficient amount of dried sample was mounted onto the SEM stub and coated with a thin layer of gold under vacuum to enhance conductivity. SEM imaging was then performed to visualize the surface topography and porous structure of the cryogels.

#### 4.3.3. FT-IR Spectroscopic Analysis

Fourier-transform infrared (FT-IR) spectroscopy was employed to identify the functional groups present in the cryogel structure. For the analysis, 2 mg of the dried cryogel sample was homogenized with 98 mg of potassium bromide (KBr) and compressed into a pellet. The FT-IR spectra were then recorded to confirm the chemical composition and successful incorporation of functional monomers.

#### 4.3.4. Elemental Analysis

Elemental analysis was conducted to quantify the incorporation of the MAPA monomer into the cryogel matrix. Approximately 1–2 mg of well-dried sample was accurately weighed and placed into the designated containers of the elemental analyzer. Since the HEMA monomer lacks nitrogen atoms while the MAPA monomer contains nitrogen, nitrogen content was used to calculate the extent of MAPA incorporation into the Poly(HEMA-MAPA) cryogel based on stoichiometric assumptions.

#### 4.3.5. Specific Surface Area (BET) Analysis

The specific surface area of the cryogel samples was determined using the Brunauer–Emmett–Teller (BET) method via nitrogen gas adsorption. Lyophilized cryogel disks were first degassed in a vacuum oven at 100 mbar and 35 °C for 6 h to remove residual moisture and gases from the pores. Subsequently, nitrogen adsorption measurements were carried out at room temperature to determine the surface area characteristics of the cryogels.

#### 4.3.6. Zeta Potential Analysis

Zeta potential measurements were performed to evaluate the surface charge of the cryogel at different pH values. Finely powdered cryogel was dispersed in 0.01 M NaCl solution and sonicated briefly. Measurements were carried out at 25 °C across a pH range of 3.0–11.0, adjusted with 0.1 M HCl or NaOH. The isoelectric point (IEP) was identified as the pH where the zeta potential approached zero.

### 4.4. Adsorption Experiments

Adsorption experiments were conducted using a batch system. Each test solution was adjusted to a total volume of 4 mL, and a pre-weighed dry cryogel disc (12.5 ± 0.5 mg) was added to initiate the adsorption process. The mixtures were incubated in sample tubes and agitated using a rotator shaker (Multi Bio RS-24, Biosan, Riga, Latvia) to ensure uniform contact between the cryogel and the MB solution. The concentration of MB in the solution before and after adsorption was measured using a UV–visible spectrophotometer (TU-1810, Pgeneral, Beijing, China) at a wavelength of 663 nm.

To optimize the adsorption conditions, experiments were performed under various parameters, including pH (3–11), contact time (5–120 min), initial dye concentration (25–5000 mg L^−1^), temperature (4–50 °C), and ionic strength (100–2000 mM NaCl). These variables were systematically varied to determine their influence on adsorption efficiency.

The adsorption capacity (*q*, mg/g) of the cryogels was calculated using the following equation:

(2) $$q=[(C_{i}-C_{f})\;x\;V]\;/\;m$$

where *C_i_* and *C_f_* are the initial and final concentrations of MB in solution (mg L^−1^), *V* is the volume of the solution (L), and *m* is the dry mass of the cryogel used (g). The capacity values are expressed as mean ± standard deviation from three independent experiments (n = 3).

### 4.5. Desorption and Reusability

The desorption behavior of MB from the cryogels was evaluated using a batch system. Following the adsorption process, the cryogels were transferred into 10 mL of a desorption medium containing 0.1 M NaOH solution and agitated continuously for 1 h to facilitate dye release.

The adsorption–desorption cycle was repeated five consecutive times using the same cryogel disc to assess the reusability of the cryogels. The cryogels were rinsed between each cycle with a 1:1 (*v*/*v*) ethanol–distilled water solution for 30 min to remove any residual dye or desorption agents. Subsequently, the cryogels were regenerated with 10 mL of distilled water (pH 7.0) for an additional 30 min before proceeding to the next cycle. This process was employed to evaluate the structural stability and regeneration efficiency of the cryogels over multiple uses.

### 4.6. Isotherms

Three different adsorption isotherm models were employed to elucidate the nature and mechanism of the adsorption interaction between MB and Poly(HEMA-MAPA) cryogels: Langmuir, Freundlich, and Flory–Huggins.

The Langmuir isotherm assumes monolayer adsorption onto a surface with a finite number of identical sites, where all adsorption sites are energetically equivalent. The linear form of the Langmuir equation is given as follows:

(3) $$\frac{1}{Q_{eq}}=\frac{1}{Q_{max}b}\frac{1}{C_{eq}}+\frac{1}{Q_{max}}$$

The Freundlich isotherm describes adsorption on heterogeneous surfaces and is expressed by the following logarithmic form:

(4) $$\ln Q_{eq}=\frac{1}{n}\ln C_{eq}+\ln K_{F}$$

The Flory–Huggins isotherm model is used to evaluate the feasibility and spontaneity of the adsorption process. It is expressed as follows:

(5) $$\log\left(\frac{\theta }{C_{eq}}\right)=\log K_{FH}+n_{FH}\log\left(1-\theta \right)$$

where

- *C*_0_ and *C_eq_* are the initial and equilibrium concentrations of MB (mg L^−1^);
- *Q_eq_* and *Q_max_* represent the equilibrium and theoretical maximum adsorption capacities (mg/g);
- *θ* is the fractional surface coverage;
- *b*, *K_F_*, *n*, *K_FH_*, and *n_FH_* are the isotherm constants.

### 4.7. Adsorption Kinetic Modeling

Kinetic modeling was performed using pseudo-first-order and pseudo-second-order kinetic equations to understand the adsorption mechanism and determine the rate-controlling steps, such as mass transfer or chemical interaction.

The pseudo-first-order kinetic model is expressed as follows:

(6) $$\log\left(q_{e}-q_{t}\right)=\log q_{e}-\left(\frac{k_{1}}{2.303}\right)t$$

where *q_e_* and *q_t_* (mg/g) are the amounts of MB adsorbed at equilibrium and at time *t*, respectively, and *k*_1_ is the rate constant of the pseudo-first-order model (min^−1^).

The pseudo-second-order kinetic model, which assumes that chemisorption is the rate-limiting step, is described by the following linearized equation:

(7) $$\frac{t}{q_{t}}=\frac{1}{k_{2}{q_{e}}^{2}}+\frac{1}{q_{e}}t$$

where *k_2_* is the pseudo-second-order rate constant (g mg^−1^ min^−1^).

### 4.8. Thermodynamic Calculations

Standard Gibbs free energy (Δ*G*°), enthalpy (Δ*H*°), and entropy (Δ*S*°) changes were calculated to gain a deeper insight into the thermodynamic nature of the adsorption process. These parameters help determine the spontaneity, heat exchange, and disorder associated with MB adsorption onto Poly(HEMA-MAPA) cryogels.

The Gibbs free energy change was calculated using the Flory–Huggins isotherm constant (*K_FH_*) according to the following equation:

(8) $${∆G}^{o}=-RT\ln K_{FH}$$

where R is the universal gas constant (8.314 J·mol^−1^·K^−1^) and *T* is the absolute temperature (*K*). The negative Δ*G*° value confirms the spontaneous nature of the adsorption process [53].

Additionally, enthalpy (Δ*H*°) and entropy (Δ*S*°) changes were derived from the Van’t Hoff equation:

(9) $$lnK=\frac{-RT}{{\Delta H}^{\circ }}+\frac{R}{{\Delta S}^{\circ }}$$

By plotting lnK against 1/*T*, Δ*H*° and Δ*S*° were calculated from the slope and intercept, respectively. The positive value of Δ*H*° indicates that the adsorption is endothermic, meaning that higher temperatures enhance MB uptake. The positive Δ*S*° suggests increased randomness at the solid–solution interface during the adsorption process, likely due to the release of water molecules and rearrangement of MB dye molecules on the hydrophobic cryogel surface [54].

## Figures and Tables

**Table 1:** Adsorption parameters.

Langmuir		
*Q_max_* (mg/g)	*b* (L/mg)	R^2^
1250	0.000093	0.9706
Freundlich		
*K_F_*	1/*n*	R^2^
0.035	1.2312	0.6877
Flory–Huggins		
*K_FH_*	*n_FH_*	R^2^
0.00015	520.62	0.7582

**Table 2:** Thermodynamic parameters of MB adsorption onto Poly(HEMA-MAPA) cryogels at different temperatures.

Temperature (K)	Δ*G*° (kJ·mol^−1^)	Δ*H*° (kJ·mol^−1^)	Δ*S*° (J·mol^−1^·K^−1^)
277	–17.89		
298	–22.02	+36.57	+196.6
323	–26.93		

**Table 3:** Kinetic modeling parameters.

Pseudo-First-Order		
*q_e_* (mg/g)	*k*_1_ (L mg^−1^)	R^2^
1066.1	0.168	0.8546
Pseudo-Second-Order		
*q_e_* (mg/g)	*k*_2 _(g^−1^ mg^−1^ min^−1^)	R^2^
1250.0	0.000356	0.9985

**Table 4:** Comparison of MB adsorption performance on various cryogel-based adsorbents.

Cryogel	Method	Isotherm Model	Adsorption Capacity (mg/g)	Equilibrium Time (min)	MB Concentration (mg L^−1^)	Ref
Poly(itaconic acid)	Batch study	Langmuir	172.4	above 400	500	[47]
AlgMA/PNaSS	Batch study	Freundlich	2300	480	192	[7]
Poly(PEGDA-co-MA)	Continuous study	Langmuir	447.7	60	1000	[48]
Poly(ionic liquids)	Batch study	Freundlich	1228.8	10	500	[49]
SA/clay quasi	Batch study	Freundlich	181.8	240	50	[50]
Poly(HEMA-MAPA)	Batch study	Langmuir	1304.6	30	2000	This work

## Data Availability

The original contributions presented in this study are included in the article. Further inquiries can be directed to the corresponding author.

## Supplementary Materials

The following supporting information can be downloaded at: https://www.mdpi.com/article/10.3390/gels11060411/s1. Figure S1: FT-IR spectrum of MAPA; Figure S2: Nitrogen adsorption isotherm of Poly(HEMA-MAPA) cryogel measured at 77 K; Figure S3: Zeta potential analysis graph for Poly(HEMA-MAPA) cryogel.
