# Supplementary material for: Amino Acid-Based Hydrophobic Cryogels for Efficient Methylene Blue Removal: A Reusable and Eco-Friendly Approach to Dye-Contaminated Wastewater Treatment
Source: Gels. 2025 May 30;11(6):411. doi: 10.3390/gels11060411 (PMC12191696; doi:10.3390/gels11060411)
Supplement: Supplementary file 1 [file gels-11-00411-s001.zip › gels-3653971-supplementary.pdf]

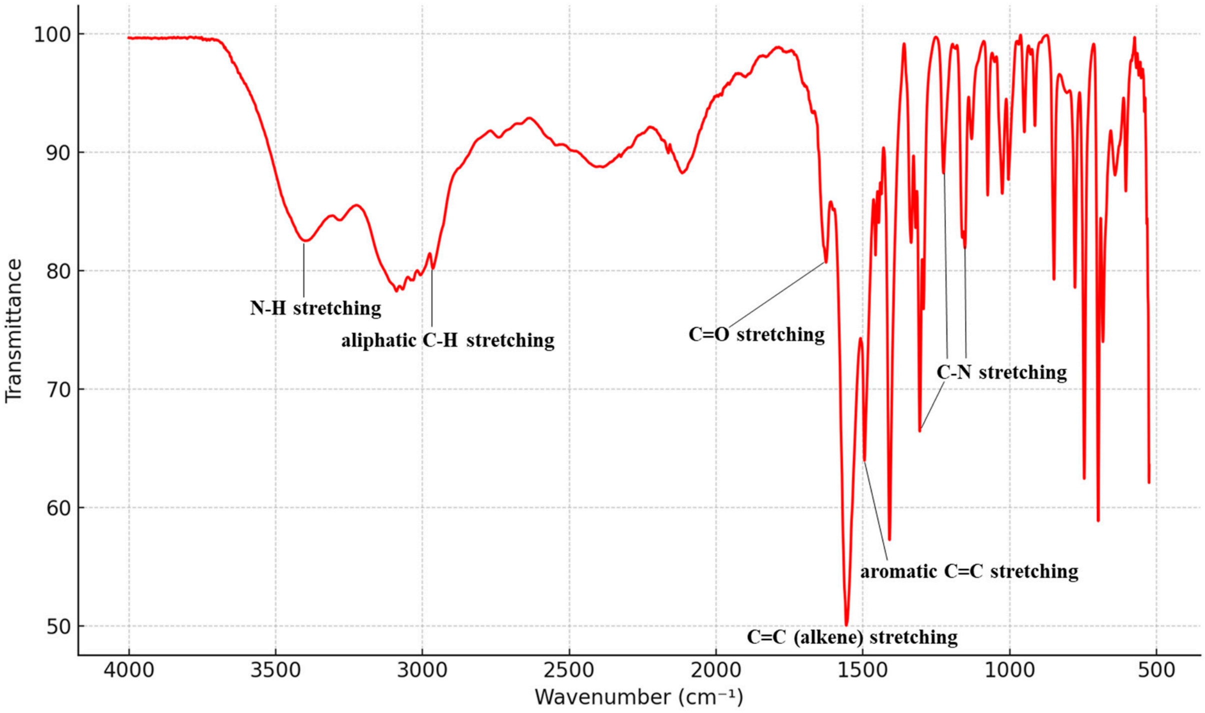

Figure S1. FT-IR spectrum of MAPA.

The FT-IR spectrum of MAPA (N-methacryloyl-L-phenylalanine) reveals characteristic absorption bands consistent with the functional groups present in its molecular structure. A broad and intense band in the region of ~3100–2500 cm^{-1} corresponds to the O–H stretching of the carboxylic acid group, while a sharp peak around ~1710–1725 cm^{-1} shows the presence of a free carboxylic acid C=O stretch. The N–H stretching vibration of the amide/amine group appears around ~3300–3400 cm^{-1}, accompanied by N–H bending near ~1600–1650 cm^{-1}. The methacryloyl moiety exhibits a C=O stretch near ~1720 cm^{-1} and a C=C stretch (alkene) between ~1635–1640 cm^{-1}. Aromatic C=C vibrations arising from the phenylalanine ring are observed within ~1450–1600 cm^{-1}. Additional aromatic ring vibrations are also evident near ~1070 cm^{-1} and ~750 cm^{-1}, further supporting the presence of the phenyl group. Aliphatic –CH_3 and –CH_2– groups show stretching bands in the ~2850–2950 cm^{-1} range. Additionally, C–N stretching bands are present between ~1200–1350 cm^{-1}, confirming amide or amine functionalities. The presence of these characteristic peaks collectively confirms the successful synthesis of MAPA.

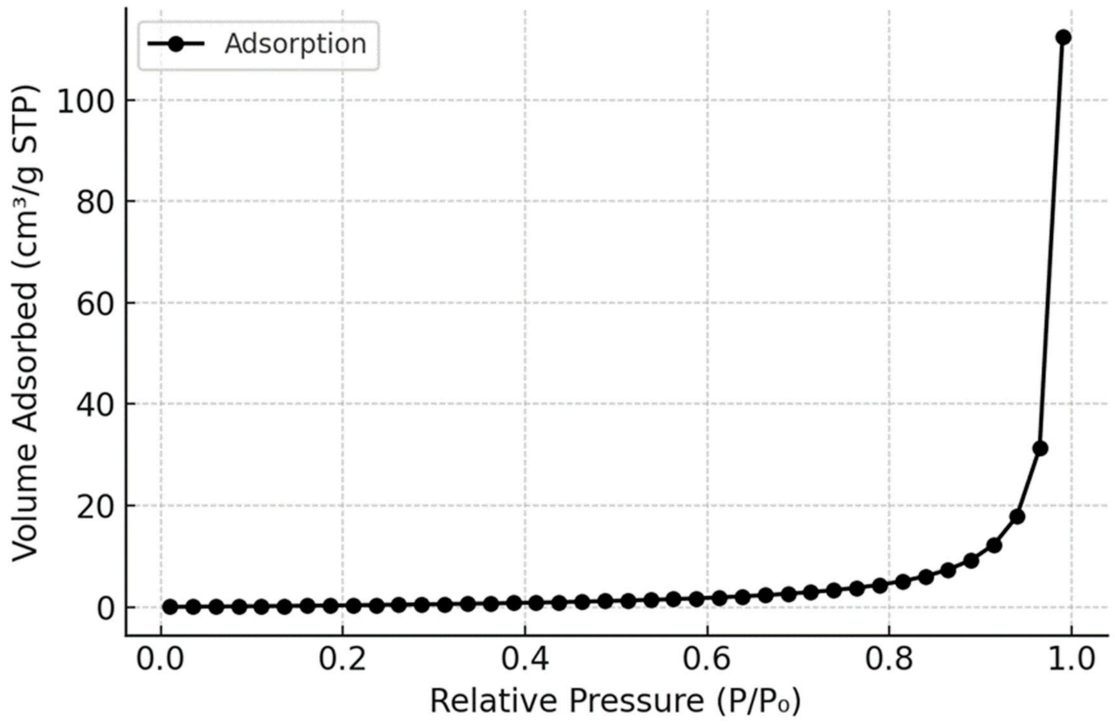

Figure S2. Nitrogen adsorption isotherm of Poly(HEMA-MAPA) cryogel measured at 77 K.

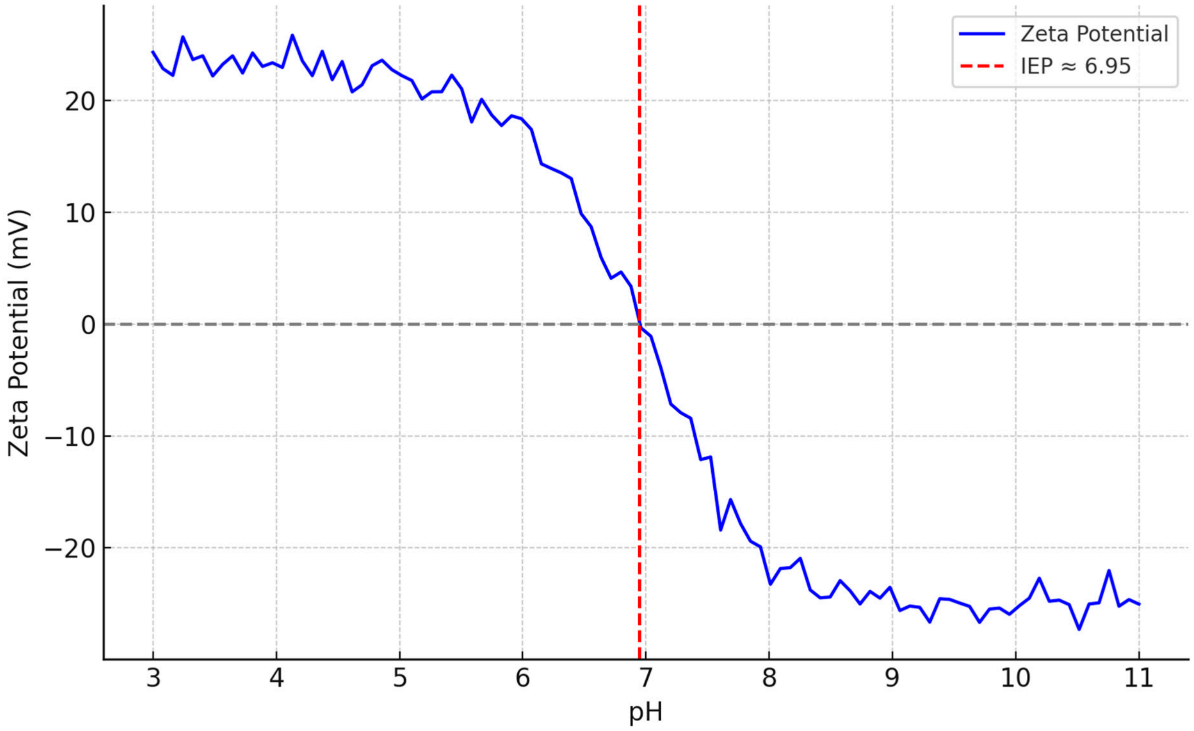

Figure S3. Zeta potential analysis graph for Poly(HEMA-MAPA) cryogel.
